# Zur gesellschaftlichen Akzeptanz von einmaligen Vermögensabgaben

**DOI:** 10.1007/s41025-021-00212-9

**Published:** 2021-02-24

**Authors:** Simon Loretz, David Stadelmann

**Affiliations:** 1 Österreichisches Institut für Wirtschaftsforschung, Wien, Österreich; 2 Universität Bayreuth, Bayreuth, Deutschland; 3 CREMA – Center for Research in Economics, Management and the Arts, Zürich, Schweiz; 4 IREF - Institute for Research in Economic and Fiscal Issues, Caluire et Cuire, Frankreich

**Keywords:** Einmalige Vermögensabgabe, Referendum, Schweiz, Gesellschaftliche Akzeptanz

## Abstract

Die Bewältigung der COVID-19 Krise verursacht erhebliche fiskalische Kosten. Um diese Budgetdefizite zu schließen, werden mitunter auch in Deutschland einmalige Steuern auf größere Vermögen gefordert. Vergangene Erfahrungen mit einmaligen Vermögensabgaben beziehen sich oft auf außergewöhnliche Nach-Kriegssituationen. Die finanziellen und gesellschaftlichen Ergebnisse solcher Abgaben waren bestenfalls gemischt. Über die breite, gesellschaftliche und politische Akzeptanz von einmaligen Vermögensabgaben ist wenig bekannt. Wir betrachten den einzigartigen, historischen Fall einer Volksabstimmung zu einer einmaligen Vermögensabgabe. Die überwältigende Ablehnung einer hohen einmaligen Vermögensabgabe in einem demokratischen Land legt nahe, dass die Bevölkerung von den Argumenten der Gegner, allen voran die Unmöglichkeit ihre Einmaligkeit zu garantieren, überzeugt wurden.

## Abstract (en)

Addressing the COVID-19 crisis imposes significant fiscal costs. In order to close these budget deficits, one-off taxes on larger wealth are sometimes demanded, lately in Germany as well. Past experience with capital levies relates to unusual post-war situations. The financial and social results of such levies have been mixed at best. The broad social and political acceptance of one-time capital levies is poorly known. We consider the unique, historical case of a referendum on a one-time capital levy. The overwhelming rejection of a large capital levy in a democratic country suggests that the population was persuaded by the opponents’ arguments, first and foremost the impossibility of guaranteeing its one-off nature.

Unmöglich kann ein demokratisches Volk einer Vermögensabgabe zustimmen, die nur wenige Besitzende erfasst und dadurch so gewaltsam das Prinzip der Allgemeinheit der Besteuerung verletzt. (Bericht des Bundesrats, 01.08.1922, BBl 1922 II 917)

## Hintergrund

**Figure 1:**
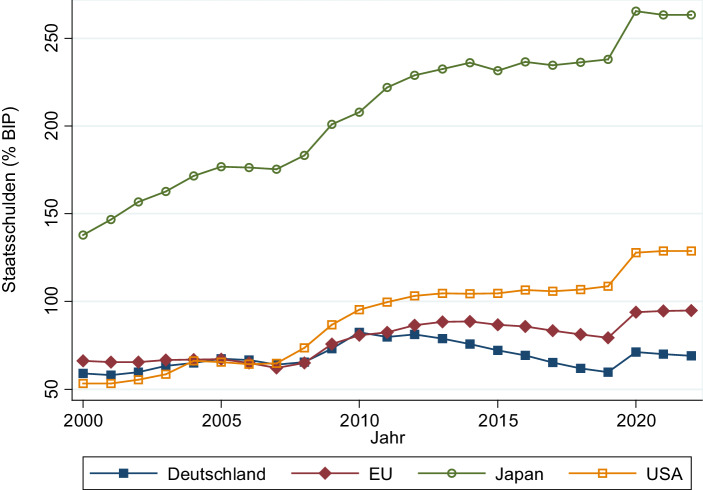

Die fiskalischen Kosten durch die COVID-19 Pandemie hinterlassen deutliche Lücken in den Staatshaushalten und verursachen einen empfindlichen Anstieg der Staatsschulden. Abb. 1 verdeutlicht den erwarteten Anstieg der Staatsschuldenquote in 2020 für Deutschland, die EU insgesamt, Japan und die USA. Dabei ist zu berücksichtigen, dass die Zunahme der Schuldenquote wie auch deren Niveau stark variieren. Japan, die USA und auch die Europäische Union insgesamt erreichen neue Höchststände in der Schuldenquote. In Deutschland könnte die Staatsschuldenquote trotz der starken Neuverschuldung aufgrund der Corona-Krise noch unter dem Niveau von 2010 bleiben.

Auf die Frage, wer die fiskalischen Kosten der COVID-19 Pandemie bezahlen soll, antworten manche politischen Akteure mit dem Vorschlag einer einmaligen Vermögensabgabe auf vermögensstarke Haushalte bzw. Unternehmen. Bereits im Zuge des Anstieges der Staatsverschuldung nach der letzten Finanzkrise kamen derartige Abgaben in wirtschaftspolitischen Debatten auf.^1^ Aktuell fordert zum Beispiel DIE LINKE in Deutschland eine progressive einmalige Vermögensabgabe.^2^ Die Forderung von einmaligen Vermögensabgaben ist nicht neu. Daher bietet sich die Möglichkeit, von anderen Ländern und aus der Geschichte zu lernen und damit offen und rational die potenziellen Vor- und Nachteile zu analysieren. Darüber hinaus kann der Frage nach der breiten gesellschaftlichen Akzeptanz von einmaligen Vermögensabgaben nachgegangen werden, die dieser Beitrag anhand einer Volksabstimmung zur Einführung einer derartigen Abgabe analysiert.

## Theoretische Überlegungen und Erfahrungen aus der Vergangenheit

In einer umfassenden Bestandsaufnahme beschreibt Eichengreen (1990) sowohl die theoretischen Rahmenbedingungen für eine erfolgreiche Umsetzung einer einmaligen Vermögensabgabe als auch die empirischen Erfahrungen von tatsächlich eingeführten Abgaben dieser Art. Im Regelfall führt eine einmalige Vermögensabgabe entgegen der landläufigen Argumentation ihrer Vertreter zu Wohlfahrtsverlusten. Eine einmalige Abgabe ist nur dann effizient und kann dem Wunsch einer schnellen Umverteilung gerecht werden, wenn (i) ihre **Einführung unerwartet** ist, sie (ii) **schnell implementiert** wird und (iii) die **glaubhafte Versicherung** gegeben werden kann, dass es sich **tatsächlich** um eine **einmalige Abgabe** handelt.

In autokratischen Systemen sind Kriterien (i) und (ii) durch eine Festsetzung der Besteuerungsgrundlage zu einem Stichtag in der Vergangenheit erreichbar. Aber auch in autokratischen Systemen besteht keine Möglichkeit, die Einmaligkeit der Abgabe glaubwürdig zu garantieren. Kempkes und Stähler (2015) modellieren eine einmalige Vermögensabgabe in einem allgemeinen Gleichgewichtsmodell und zeigen, dass die Erwartungen an eine wiederholte Verwendung der Abgabe zu negativen Wohlfahrtseffekten führen. Selbst durch Versicherung eines zukünftig nachhaltigen Staatshaushalts, und selbst wenn eine wiederholte Einhebung einer einmaligen Vermögensabgabe glaubhaft mit hohen politischen Kosten verbunden ist, kann nach Einführung die Erwartungshaltung für zukünftige „einmalige“ Vermögensabgaben nicht vollständig ausgeschlossen werden. Dadurch werden dauerhaft Ausweichreaktionen stimuliert.^3^

In demokratischen Ländern mit klaren Verfassungsrechten ist eine rückwirkende Festlegung einer Vermögensabgabe nahezu ausgeschlossen, genauso wie die schnelle Implementierung im parlamentarischen Prozess. Laut einem verfassungsrechtlichen Gutachten des wissenschaftlichen Dienstes des Deutschen Bundestages (2008, S. 11) setzt die Einhebung einer einmaligen Vermögensabgabe eine „[…] existenzbedrohende finanzielle Notlage des Staates voraus, in der weder eine Steigerung der Einnahmen aus den übrigen Steuern noch eine Ausweitung der Kreditaufnahme oder eine entsprechende Ausgabenkürzung möglich sei“. Im Zusammenhang mit der Corona-Pandemie wurde der wissenschaftliche Dienst des Deutschen Bundestages (2020, S. 6) erneut mit einem Gutachten beauftragt, und kommt zum Schluss, dass sich eine einmalige staatliche Ausnahmesituation aufgrund der aktuellen Corona-Pandemie zum jetzigen Zeitpunkt jedenfalls „noch nicht abschätzen“ lässt und mit den historischen Ereignissen, welche den Wehrbeitrag von 1913, das Reichsnotopfer von 1919 und die Lastausgleichsabgabe von 1952 begründet haben, „wohl nicht vergleichbar“ ist.^4^ Die oben angeführte Abb. 1 würde diese Position wenigstens für Deutschland stark unterstützen: Die Staatsschuldenquote liegt (noch) unter jener während der Finanzkrise. Klar ist jedenfalls, dass in einem demokratischen Staat mit geltender Verfassung eine einmalige Vermögensabgabe kaum schnell und unerwartet eingeführt werden kann.

Grundsätzlich zeigen Erfahrungen aus der Vergangenheit, dass es nur wenige potenziell gesamtgesellschaftlich positive Umsetzungen von einmaligen Vermögensabgaben gegeben haben dürfte. Nur die Vermögensabgabe in den Jahren 1946–47 in Japan kann nach Eichengreen (1990), dank der Unterstützung durch die amerikanische Besatzung und der außergewöhnlichen politischen Umstände direkt nach dem Krieg, als erfolgreich angesehen werden. Vermögensabgaben in Italien im Jahr 1920 und der Lastenausgleichsfond in Deutschland im Jahr 1952 konnten über lange Zeiträume bezahlt werden und hatten damit im Grunde den Charakter einer erweiterten Kapitalertragssteuer. Sie erwiesen sich nach den Analysen von Bach et al. (2014) als relativ erfolgreich.^5^ Inwiefern der deutsche Lastenausgleich nach den Erfahrungen der Diktatur und der damaligen Situation breit gesellschaftlich unterstützt, oder – wie vieles davor und in der Zeit – geduldet wurde, ist unklar. In der Corona-Pandemie wurde kein physisches Kapital zerstört wie im zweiten Weltkrieg. Das Vertrauen in den Staat ist ebenfalls noch hoch und nicht durch unverhältnismäßige Politikinterventionen gefährdet.

Die Vermögensabgabe im Zuge des Lastenausgleichs in Deutschland bezog sich primär auf Immobilienvermögen, welches den Weltkrieg unbeschadet überstanden hatte. Im Gegensatz dazu bezieht sich die Diskussion zu Vermögensabgaben derzeit im Regelfall auf das Gesamtvermögen, welches zu einem Stichtag bewertet werden soll. Bei Beteiligungsvermögen verändert sich das Vermögen ex-post oft beträchtlich. In der aktuell unsicheren wirtschaftlichen Lage mit großen erwarteten Strukturanpassungen führt eine Festlegung der Bemessungsgrundlage zu einem Stichtag in der Vergangenheit direkt auch zu unerwünschten Ergebnissen: Zum Beispiel haben Aktienwerte in von der Corona-Krise hart getroffenen Branchen seit dem 01.01.2020 deutlich an Wert verloren,^6^ womit eine einmalige Vermögensabgabe zu diesem Stichtag die Geschädigten durch die Corona-Krise relativ stärker belasten würde, als Personengruppen, die seit Ausbruch der Krise ihr Vermögen vermehrt haben.

Bei heute sehr mobilem Kapital sind einerseits schnelle Ausweichreaktionen möglich. Andererseits wäre es schwieriger möglich als in der Vergangenheit, als Kapital beispielsweise nach dem Weltkrieg knapp war, nachhaltig hohe Kapitalrenditen zu erwirtschaften, um die Kosten der Vermögensabgabe zu tragen. Eine Überwälzung einer Vermögensabgabe auf andere Produktionsfaktoren wie Arbeit ist daher naheliegender. Darüber hinaus ist heute von keiner nennenswerten Inflation auszugehen. Somit wäre die reale Belastung einer einmaligen Vermögensabgabe unter den derzeitigen makroökonomischen Rahmenbedingungen deutlich höher.^7^

Wie die Akzeptanz von einmaligen Vermögensabgaben in stabilen, etablierten Demokratien ist, konnte bis jetzt keine Studie evaluieren, da die allgemeine Zustimmung der Bevölkerung zu einer derartigen steuerlichen Maßnahme in der Regel nicht erhoben wird.

## Erfahrungen aus der Schweiz

Die tatsächliche Zustimmung zu einer einmaligen Vermögensabgabe lässt sich wenigstens an einem Fallbeispiel aus der Schweiz analysieren. Die Schweiz hat eine ausgeprägte direkte Demokratie. Dies ermöglicht es uns, die breite gesellschaftliche Akzeptanz der Bevölkerung im Rahmen eines demokratischen Prozesses zu evaluieren. Entscheidungen in Volksabstimmungen reflektieren die Präferenzen der Bürger für politische Alternativen: Entweder die Referendumsvorlage wird angenommen oder der Status quo bleibt bestehen (vgl. Stadelmann et al. 2013).

Wir greifen auf eine Volksabstimmung zu einer Initiative im Jahr 1922 zurück, welche eine einmalige Vermögensabgabe über eine Verfassungsänderung forderte. Wir evaluieren damit die Zustimmung zu einer einmaligen Vermögensabgabe in der breiten Bevölkerung auf eine direkte und einfach nachvollziehbare Weise. Die Ergebnisse zeigen, dass die einmalige Vermögensabgabe auf die vermögendsten Bürger auf keine breite gesellschaftliche Zustimmung stieß.

Auf die ursprüngliche Initiative der sozialdemokratischen Partei wurde in der Schweiz am 3. Dezember 1922 eine Volksabstimmung über eine einmalige Vermögensabgabe abgehalten. Vor dem Hintergrund der schwierigen Wirtschaftslage in der Schweiz und der hohen Staatsverschuldung wurde eine einmalige Besteuerung von Vermögen gefordert. Sie sollte dazu beitragen, den Staatshaushalt zu konsolidieren. Damit ist das Ziel der Abgabe ähnlich zu jenem der heutigen Diskussionen über eine einmalige Vermögensabgabe.

Im Speziellen sieht der Vorschlag eine einmalige Vermögensabgabe auf das Gesamtvermögen (zum Stichtag des 31.12.1922 und damit nicht rückwirkend) von natürlichen und juristischen Personen vor (vgl. Anhang). Der progressive Steuersatz steigt von 8 % auf Vermögen über 80.000 Franken bis 60 % für Vermögen von über 32,7 Mio. Franken. 80.000 Schweizer Franken im Jahr 1922 entsprechen heute ca. 2.100.000 Schweizer Franken (etwa 1.930.000 €),^8^ und nach Schätzungen des Schweizer Bundesrats hätte die Vermögensabgabe nur etwa 0,6 % der Schweizer Bevölkerung betroffen.

Im Verfassungstext zur Abstimmung war explizit geregelt, dass es sich de jure um eine einmalige Vermögensabgabe handelt. Der Verfassungsartikel wäre nach einmaliger Erhebung wieder gestrichen worden.^9^ Somit sind wenigstens auf dem Papier die Bedingungen für eine effiziente Steuer vergleichsweise gut gegeben: Nach Abstimmung wird die Steuer beinahe sofort bindend und der Einmal-Charakter ist verfassungsrechtlich zugesichert. Da die Abgabe zusätzlich nur 0,6 % der Bevölkerung betrifft, könnte man erwarten, dass die Zustimmung für dieses Referendum beträchtlich ausfallen hätte sollen, denn der Großteil der Bevölkerung wäre wenigstens nicht direkt betroffen gewesen.

Aus Tab. 1 ist ersichtlich, dass die einmalige Vermögensabgabe mit 87 % Nein-Stimmen im Referendum äußerst deutlich abgelehnt wurde. Gleichzeitig war die Wahlbeteiligung mit 86,3 % die höchste, die jemals in der Schweiz beobachtet wurde.^10^ Die einmalige Vermögensabgabe scheint damit für die breite Bevölkerung gesellschaftlich nicht akzeptabel gewesen zu sein. Die Vorlage wurde klar verworfen. Uns ist historisch und aus verschiedenen Ländern kein anderer Fall bekannt, in dem eine breite gesellschaftliche Ablehnung einer Vermögensabgabe auf so direkte und klare Weise ersichtlich wird. Die Ausgestaltung der geforderten Verfassungsänderung und die Situation zum damaligen Zeitpunkt machen die Abstimmung zu einem lehrreichen Fall.

**Table 1:** 

	Wahlbeteiligung		Wahlergebnis	
	Absolut	In Prozent	Absolut	In Prozent
*Stimmberechtigte:*	992.523	100	–	–
*Abgegebene Stimmen:*	856.148	86,3	–	–
Davon leer:	7525	0,8	–	–
Ungültig:	1969	0,2	–	–
Gültig:	846.654	85,3	846.654	100
**Ja:**	–	–	**109.702**	**13,0**
**Nein:**	–	–	**736.952**	**87,0**

Die statistisch plausibilisierten Ergebnisdaten des Bundesamts für Statistik können von den amtlich verbindlichen Ergebnissen der Bundeskanzlei leicht abweichen

Quelle: Eigene Berechnungen auf Basis der Ergebnisdaten des Bundesamts für Statistik (Schweiz)

**Figure 2:**
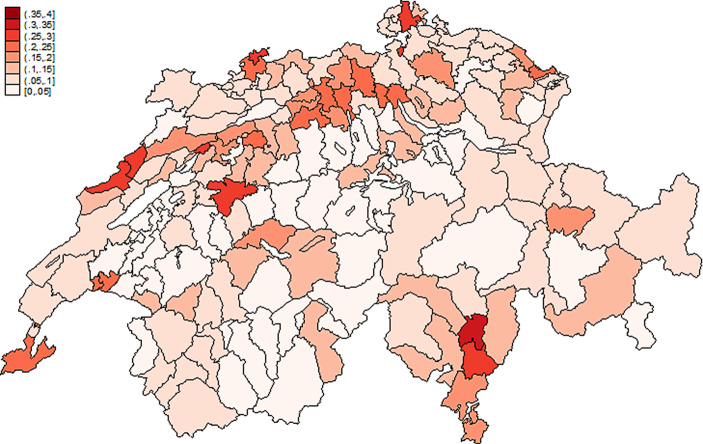

Abb. 2 verdeutlicht, dass es trotz der klaren Gesamtablehnung gewisse geographische Variation in den Ergebnissen gab. Zwar lehnten alle Kantone und auch alle Bezirke die einmalige Vermögensabgabe ab. Doch im relativ kleinen Bezirk Riviera im Tessin stimmten immerhin fast 35 % der Wahlberechtigten für die einmalige Vermögensabgabe.^11^ Am anderen Ende des Spektrums finden sich viele Wahlbezirke in der Zentralschweiz mit Zustimmungsraten von deutlich unter 5 %.

Auf kantonaler Ebene erhielt die Initiative in Basel Stadt (27,6 %) und Genf (24,8 %) zwar ebenfalls eine klare Ablehnung aber doch eine etwas größere Unterstützung als in den zentralen Kantonen Obwalden (1,9 %) und Nidwalden (1,9 %). In den damals vergleichsweise armen Kantonen Nidwalden und Appenzell Innerrhoden stimmten absolut sogar nur 57 und 66 einzelne Bürger für die einmalige Vermögensabgabe. Die existierende Variation in der Ablehnung der Vorlage kann für weitere Analysen genutzt werden.

## Erklärungen für gesellschaftliche Ablehnung von einmaligen Vermögensabgaben

Das Fallbeispiel zeigt, dass eine überwältigende Mehrheit in einem demokratischen Land eine einmalige Vermögensabgabe ablehnte, von welcher sie nicht direkt negativ betroffen war, die die Gesamtschulden reduzieren hätte können, die nur die sehr Vermögenden belastet hätte und wenigstens scheinbar vergleichsweise effizient hätte sein können. Wie lässt sich dies erklären?

**Figure 3:**
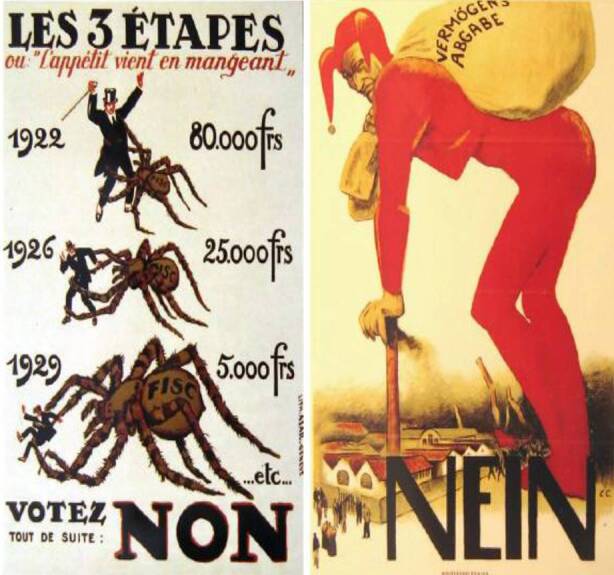

Die Gegner der Vermögensabgabe hatten Argumente, von denen sie weite Teile der Bevölkerung überzeugen konnten. Die Überzeugungsarbeit wurde einerseits, ähnlich wie auch heute, über emotionale Plakat-Kampagnen geführt. Sie stellten die theoretisch wesentlichen Nachteile einer einmaligen Vermögensabgabe visuell dar. Abb. 3 zeigt exemplarisch zwei Plakate der Gegner, welche die Last, die eine Vermögensabgabe darstellt, veranschaulichen. Die Plakate spielen auch auf den Enteignungscharakter solcher Abgaben und auf die Unglaubwürdigkeit des Einmal-Charakters an. Beim Schweizer Sozialarchiv finden sich zahlreiche weitere Plakate, welche zusätzlich noch den zu erwartenden Verwaltungsaufwand, eine drohende Inflation und weiter steigende Steuern aufgrund eines wirtschaftlichen Einbruchs thematisieren. Dem Gegenüber finden sich nur sehr vereinzelt Plakate der Befürworter, welche z. B. die einmalige Vermögensabgabe in Verbindung mit der Alters- und Invalidenversicherung setzen.

Nicht plakativ, dafür verbal und analytisch deutlich werden die Probleme bis hin zur Ungerechtigkeit einer solchen Abgabe im Bericht des Schweizer Bundesrats zur Initiative dargelegt. Obgleich die Volksabstimmung fast 100 Jahre zurückliegt und die Initiative teilweise höhere Steuersätze beinhaltet als derzeit diskutierte Vorschläge, so haben die verwendeten Argumente der damaligen eidgenössischen Regierung noch heute Gültigkeit und Relevanz.

Zunächst wird auf die Verletzung des Postulates der **Allgemeinheit einer Steuer** hingewiesen, welches fordert, dass Steuern grundsätzlich alle treffen sollten. Während dies nicht ausschließt, sehr kleine Vermögen nicht zu belasten, ist eine Steuer, welche fundamental nur auf eine sehr kleine Gruppe abzielt nicht als allgemein zu betrachten. Oder mit den Worten des Bundesrates der Schweizerischen Eidgenossenschaft (1922, S. 950): „*Eine Steuer, die nur 6* *‰ der Bevölkerung trifft, ist in der Demokratie unzulässig. Indem sie sich demokratisch gebärdet, zielt die Initiative auf einen Ausnahmezustand ab, der den, unsern demokratischen Einrichtungen zugrunde liegenden Grundsätzen der Gleichheit widerspricht*“.

Zusätzlich wird kritisiert, dass niemals verhindert werden kann, „[…] *dass zwei scheinbar gleiche Vermögen, die aber ihren Besitzern sehr verschiedene Einkünfte gewähren, demselben Steuersatze unterliegen*“ (ibid. S. 924). Damit wird direkt angesprochen, dass eine Substanzsteuer gegen das **Leistungsfähigkeitsprinzip** verstößt. Der bloße Besitz von Vermögen muss nicht bedeuten, dass eine Person dadurch mehr Einkommen erzielen kann und somit in der Lage ist, eine höhere Steuerlast zu schultern. Die schweizerische Regierung verdeutlicht dies noch zusätzlich: „*Den Rentner, der bereits unter der Verteuerung der Lebenskosten und den Verlusten leidet und dessen vorgerücktes Alter die Bestreitung des Lebensunterhalts aus eigener Arbeit verbietet, bringt die Vermögensabgabe in grosse Verlegenheit*“ (ibid. S. 925).

Daher ist es auch verfehlt, Vermögensabgaben allgemein – und **einmalige** Vermögensabgaben im Speziellen – isoliert zu betrachten, was einen weiteren theoretischen Ablehnungsgrund einer einmaligen Vermögensabgabe darstellen dürfte. Noch naheliegender und deutlich wichtiger als die Berücksichtigung der persönlichen Lebensumstände ist damit die **integrierte Betrachtung **der Vermögensabgabe im Kontext der gesamten Abgabenbelastung und der staatlichen Leistungen (Äquivalenzprinzip). Auch der Bundesrat (1922, S. 927) hält fest, „*da die Vermögensabgabe sich zu den andern Steuern gesellt, gilt es zu erfahren, in welchem Masse das ganze Steuersystem den Pflichtigen zur öffentlichen Beitragsleistung heranzieht*“. Die gesamtheitliche Betrachtung des Steuersystems wäre imperativ bei der Diskussion von jeglichen Arten von Vermögensabgaben. Die Doppelbelastung von Kapital durch Substanzsteuern zusätzlich zu Kapitalertragssteuern führt in der Regel zu einer hohen Gesamtsteuerbelastung des Faktors Kapital. Dies kann zur Folge haben, dass Kapital entweder abwandert, die Sparquoten derartig sinken, dass sich die Kapitalmenge reduziert, die Steuerbelastung auf Arbeit überwälzt wird und der gesamtwirtschaftliche Wohlstand aller Bevölkerungsgruppen abnimmt.

Ein auf die **Berechnung des Vermögens** zu einem Stichtag zurückzuführendes Problem ist, dass der Fall auftreten kann, „[…] *dass der Steuerpflichtige eine Steuer für ein Vermögen zu entrichten hat, das nicht mehr vorhanden ist, während die inzwischen neu erworbenen Vermögen von der Abgabe befreit bleiben*“ (ibid. S. 926). Dieses Argument zeigt, wie in den o. a. theoretischen Überlegungen zuvor dargestellt, dass auch eine rückwirkende Festlegung der Vermögensabgabe zu relevanten Problemen führt und wohl nur schwer als „gerecht“ bezeichnet werden kann.

Obgleich man die Argumente der Gegner der Abstimmung zur einmaligen Vermögensabgabe sowie jene des Bundesrates nicht teilen muss und auch die Präferenzen des Schweizer Stimmvolks zum damaligen Zeitpunkt anders als heute ansehen könnten, erscheint es erstaunlich, dass die heutigen Befürworter von einmaligen Vermögensabgaben auf die damaligen Bedenken, die auch heute noch gelten, wenig entgegen bringen. Noch erstaunlicher ist höchstens, dass die heutigen Gegner von einmaligen Vermögensabgaben nicht einfach die damaligen Argumente übernehmen und geringfügig anpassen. Wir spekulieren, dass die Abstimmung in der Schweiz einfach weitgehend unbekannt ist.

## Empirische Analyse des Abstimmungsverhaltens

Ob es die eindrückliche Plakat-Kampagne, die Argumente des Bundesrates oder die individuellen Abwägungen der Abstimmenden waren, die zu einer klaren Ablehnung der einmaligen Vermögensabgabe führten, ist rund 100 Jahre nach der Abstimmung nicht mehr zu eruieren. Wohl aber können potenzielle Determinanten des Abstimmungsverhaltens mittels multipler Regressionsanalyse beleuchtet werden. Wir haben dazu Daten um das Jahr 1922 für die einzelnen Kantone aufgearbeitet^12^ und präsentieren in Tab. 2 Resultate.^13^

**Table 2:** 

	Zu erklärende Variable: Anteil der Ja-Stimmenden									
	(1)	(2)	(3)	(4)	(5)	(6)	(7)	(8)	(9)	(10)
SP Wähler	0,443*** (0,079)	–	–	–	–	–	–	–	–	0,168 (0,203)
Wahlbeteiligung	–	−0,473** (0,188)	–	–	–	–	–	–	–	−0,340*** (0,092)
Bevölkerungsdichte	–	–	0,055*** (0,010)	–	–	–	–	–	–	0,212*** (0,060)
Anteil von Katholiken	–	–	–	−0,126*** (0,034)	–	–	–	–	–	−0,042 (0,136)
Firmendichte	–	–	–	–	0,004** (0,002)	–	–	–	–	0,037 (0,103)
Anteil Großgrundbesitzer	–	–	–	–	–	−0,134*** (0,034)	–	–	–	−0,360** (0,156)
Anteil Fabriksarbeiter	–	–	–	–	–	–	0,549 (0,360)	–	–	0,172* (0,093)
Anteil Arbeitssuchende	–	–	–	–	–	–	–	0,193** (0,071)	–	0,064 (0,086)
Kantonale Anleihen pro Kopf	–	–	–	–	–	–	–	–	0,168* (0,096)	0,343* (0,176)
Konstante	0,030*** (0,012)	0,517*** (0,164)	0,095*** (0,012)	0,180*** (0,024)	0,034 (0,031)	0,156*** (0,020)	0,068*** (0,027)	0,082*** (0,015)	0,061*** (0,023)	0,000 (0,087)
Korrigiertes R^2^	0,529	0,181	0,303	0,299	0,127	0,155	0,120	0,162	0,301	0,810
Beobachtungen	25	25	25	25	25	25	25	25	25	25

Robuste Standardfehler in Klammern. In (10) werden standardisierte beta-Koeffizienten berichtet

Quelle: Eigene Berechnungen

***p < 1 %, **p < 5 %, *p < 10 %

Die naheliegendste erklärende Variable wäre der Stimmenanteil der Sozialdemokratischen Partei der Schweiz, welche die Volksabstimmung initiiert hatte. Die Nationalratswahl fand am 29. Oktober 1922 und somit nur knapp einen Monat vor dem Referendum über die einmalige Vermögensabgabe statt. Bundesweit erzielte die sozialdemokratische Partei 23,3 % der Stimmen. Ein Vergleich mit dem Ja-Stimmen-Anteil für die einmalige Vermögensabgabe von 13 % zeigt bereits, dass die Sozialdemokraten nicht in der Lage waren, alle ihre eigenen Wähler zu überzeugen: Selbst unter der Annahme, dass alle Ja-Stimmen von Wählern der sozialdemokratischen Partei kamen, wäre die Zustimmung mit 109.702 substantiell unter der Wählerzahl der Sozialdemokraten bei der Nationalratswahl von 170.974. Das Regressionsergebnis in Spalte (1) in Tab. 2 verdeutlicht, dass ein 1 Prozentpunkt höherer Wähleranteil der Sozialdemokraten sich nur in 0,44 Prozentpunkten höherer Zustimmung zur einmaligen Vermögensabgabe niedergeschlagen haben könnte. Somit gab es – durchschnittlich betrachtet – keine mehrheitliche Zustimmung bei den Wählern der Partei, welche den Vorschlag eingebracht hat. Von einer breiten Unterstützung selbst innerhalb der sozialdemokratischen Partei zu einmaligen Vermögensabgaben kann nicht gesprochen werden: Die Parteiführung vertrat insofern mit hoher Wahrscheinlichkeit nicht einmal die Hälfte ihrer eigenen Wähler.

Eine selektiv geringere Wahlbeteiligung potenzieller Befürworter kann ein möglicher Erklärungsansatz für die geringe Zustimmung sein. Der hoch signifikante negative Koeffizient in Spalte (2) bestätigt dies grundsätzlich. Dabei ist jedoch zu beachten, dass generell die Wahlbeteiligung sehr hoch war, und somit nur ein geringer Teil der Variation der Abstimmungsergebnisse erklärt werden kann.^14^

Als jeweilig alleiniger Bestimmungsgrund herangezogen, tragen sowohl die Bevölkerungsdichte (Spalte 3), die Firmendichte (Spalte 5), der Anteil der Fabriksarbeiter (Spalte 6, nicht statistisch signifikant), und der Anteil der Arbeitssuchenden (Spalte 8) zu einer tendenziell größeren Zustimmung bei, während in den Kantonen mit größerem Anteil an Katholiken (Spalte 4) und mehr landwirtschaftlichen Großgrundbesitzern (Spalte 6) deutlich weniger Zustimmung beobachtet werden kann.^15^ Die größere Zustimmung in den urbanen Gebieten bzw. in Kantonen mit höherer Firmendichte misst vermutlich einen ähnlichen Effekt wie der höhere Anteil an Fabriksarbeitern. Ähnlich gibt es einen Zusammenhang zwischen der breiten Ablehnung in den katholisch geprägten Kantonen und dem größeren Anteil an landwirtschaftlichen Grundbesitzern. Zusätzlich gibt es einen stark negativen Zusammenhang zwischen der katholischen Prägung und dem Stimmenanteil der Sozialdemokraten. Eine nur leicht höhere Zustimmung gibt es in Kantonen mit höherer Verschuldung (Spalte 9).

Um die Wirkungen der einzelnen Bestimmungsfaktoren miteinander vergleichbar zu machen, inkludiert Regression (10) in Tab. 2 alle Variablen gleichzeitig. Zusätzlich sind alle Variablen transformiert, um die Effektstärke vergleichbar zu machen. Der stark negative Effekt für die Wahlbeteiligung bleibt bestehen, während sich kein Zusammenhang mehr für den Stimmenanteil der Sozialdemokratischen Partei zeigt. Die Bevölkerungsdichte und der Anteil der Fabriksarbeiter im Kanton tragen ebenfalls statistisch signifikant positiv zu einem höheren Zustimmungsgrad bei, was aus einer klassenkämpferischen Sicht heraus leicht erklärbar ist. Die überdurchschnittliche Ablehnung in den Kantonen mit mehr landwirtschaftlichem Großgrundbesitz mag ebenso nachvollziehbar sein, wie eine größere Zustimmung in Kantonen mit höherem kantonalen Verschuldungsgrad. Für den Anteil der Katholiken, den Anteil an Arbeitssuchenden und die Firmendichte im Kanton findet sich hingegen kein signifikanter Effekt mehr.

Insgesamt deuten die Ergebnisse darauf hin, dass in urbanen Gebieten mit Industrie die gesellschaftliche Akzeptanz von einmaligen Vermögensabgaben etwas höher ist als in ländlichen großbäuerlich geprägten Gebieten. Ein deutlich negativer Zusammenhang zwischen dem Ja-Stimmen-Anteil und der Wahlbeteiligung zeigt die stärkere Mobilisierung der Gegner. Obwohl die Zustimmung wie erwartet deutlich stärker in den Kantonen mit einem höheren sozialdemokratischen Wähleranteil war, hat dieser Wähleranteil keinen zusätzlichen Erklärungsgehalt, wenn für die anderen Faktoren kontrolliert wird. Dabei sei nochmals darauf hingewiesen, dass die Zustimmung für alle Kantone weit unter einem Drittel liegt und die Bevölkerung insgesamt eine einmalige Vermögensabgabe stark abgelehnt hat.

## Schlussfolgerungen

Diskussionen über eine einmalige Vermögensabgabe zur schnelleren Konsolidierung von Staatsschulden werden oft nach und während größeren und kleineren Krisen geführt. Ob eine breite, gesellschaftliche Akzeptanz für derartige Abgaben existiert, ist schwierig zu messen, da die Bürger im Regelfall nicht bei Abgabenentscheidungen systematisch und unverzerrt befragt werden bzw. selbst mitstimmen können.

Ein systematischer Blick auf einen Fall aus der Vergangenheit zeigt eine minimale gesellschaftliche Akzeptanz zu einmaligen Vermögensabgaben. Wir analysieren eine Volksabstimmung in der Schweiz in 1922, in der die Bevölkerung über eine einmalige Vermögensabgabe entschieden hat. Die einmalige Vermögensabgabe wurde von einer breiten Bevölkerungsschicht eindeutig und bei hoher Abstimmungsbeteiligung abgelehnt. Uns ist kein anderer historischer Fall bekannt, der eine so gezielte Analyse gesellschaftlicher Akzeptanz einer Vermögensabgabe erlaubt.

Die Situation in der Schweiz 1922 kann durchaus als drastischer angesehen werden, als die Krisen der letzten Jahre. Gleichzeitig lässt sich eine historische Präferenzbezeugung nicht direkt auf ein anderes Land zu einer anderen Zeit projizieren.^16^ Dennoch zeigt die außerordentlich deutliche Ablehnung, dass die Argumente der Gegner Gehör fanden. Viele der damals vorgebrachten Argumente gegen eine einmalige Vermögensabgabe haben in einem demokratischen Land auch heute noch ihre fundamentale Gültigkeit. Eine Abgabe, welche gezielt einen kleinen Teil der Bevölkerung trifft, dafür aber umso massiver, ist mit dem Grundsatz der Allgemeinheit der Besteuerung nicht vereinbar. Zusätzlich verstößt die Besteuerung der Vermögenssubstanz gegen das Leistungsfähigkeitsprinzip und kann/soll nicht ohne Berücksichtigung der gesamten Steuerlast diskutiert werden. Besonders wesentlich ist, dass eine einmalige Vermögensabgabe nicht glaubhaft einmalig ist und damit reale, potenziell hohe Wohlfahrtsverluste drohen. In der Schweiz in 1922 wurde die Vorlage einer einmaligen Vermögensabgabe in einem demokratischen Volksentscheid mit 87,0 % klar abgelehnt. Da die heutigen Vermögen mobiler sind als in der Vergangenheit und die heutige Bevölkerung breiter gebildet ist, darf spekuliert werden, dass die Gegenargumente durchaus auf noch breiteres Verständnis stoßen könnten. Jedenfalls sollte der historische Fall den Vertretern von oft *wiederholten* Forderungen nach *einmaligen* Vermögensabgaben, welche aktuell eine gewisse Renaissance erleben, etwas zu denken geben.

## Anhang

### Originalwortlaut der vorgeschlagenen Verfassungsänderung

1. Der Bund erhebt eine einmalige Vermögensabgabe zu dem Zwecke, sich, den Kantonen und den Gemeinden die Erfüllung der sozialen Aufgaben zu ermöglichen.
2. Abgabepflichtig sind die natürlichen und die juristischen Personen.
3. Von der Entrichtung der Abgabe sind befreit:
  - der Bund und die Kantone und ihre Anstalten und Betriebe sowie die unter ihrer Verwaltung stehenden Spezialfonds, die Schweizerische Nationalbank, die Schweizerische Unfallversicherungsanstalt und die Schweizerische Alkohol Verwaltung;
  - die Gemeinden sowie die andern öffentlich-rechtlichen und kirchlichen Körperschaften und Anstalten für das Vermögen, das als solches oder mit seinem Ertrag öffentlichen Zwecken dient;
  - die übrigen Körperschaften und Anstalten für das Vermögen, das als solches oder mit seinem Ertrag Kultus oder Unterrichtszwecken oder der Fürsorge für Arme und Kranke sowie für Alter und Invalidität oder andern ausschliesslich gemeinnützigen Zwecken dient.
4. Abgabepflichtig ist das gesamte Vermögen nach Abzug der Schulden. Vorbehalten bleiben die Bestimmungen der Ziffern 5, 6 und 9.
5. Als abgabepflichtiges Vermögen natürlicher Personen gilt nicht der Hausrat bis auf einen Betrag von 50.000 Fr.
6. Als abgabepflichtiges Vermögen juristischer Personen gelten nicht:
  - das einbezahlte Grund- oder Stammkapital,
  - die Rücklagen für ausschliesslich gemeinnützige oder Wohlfahrtszwecke, deren Verwendung zu solchen Zwecken gesichert ist.
7. Für die Veranlagung der Vermögensabgabe wird das Vermögen von Ehegatten, die nicht dauernd voneinander getrennt leben, zusammengerechnet.
8. Für die persönliche und sachliche Abgabepflicht und die Einschätzung ist der 31. Dezember 1922 als Stichtag massgebend.
9. Abgabepflichtig ist bei natürlichen und juristischen Personen nur der den Betrag von 80.000 Fr. übersteigende Teil des Vermögens.
  Der abgabefreie Betrag erhöht sich bei Familien: *a)* für die Ehefrau um 30.000 Fr.; *b)* für jedes minderjährige Kind um 10.000 Fr.
10. Für die natürlichen Personen beträgt die Vermögensabgabe für die ersten angefangenen oder vollen
  **Table:**
  	Fr		vom Hundert
  für die ersten angefangenen oder vollen	50.000	des abgabepflichtigen Vermögens	8
  für die nächsten angefangenen oder vollen	50.000	″	10
  ″	100.000	″	12
  ″	200.000	″	14
  ″	300.000	″	16
  ″	400.000	″	18
  ″	600.000	″	20
  ″	1.000.000	″	22
  ″	1.000.000	″	24
  ″	1.000.000	″	26
  ″	2.000.000	″	28
  ″	2.000.000	″	30
  ″	2.000.000	″	32
  ″	2.000.000	″	34
  ″	2.000.000	″	37
  ″	2.000.000	″	40
  ″	2.000.000	″	43
  ″	3.000.000	″	46
  ″	3.000.000	″	49
  ″	3.000.000	″	52
  ″	3.000.000	″	56
  ″	für alle weiteren Beträge …		60
  11. Die Vermögensabgabe ist vom 1. Januar 1923 an mit 6 vom Hundert zu verzinsen.
  12. Die Vermögensabgabe kann in einem Betrage oder innert drei Jahren in jährlichen Tilgungsraten entrichtet werden.
  13. Nachweislich selbst gezeichnete Obligationen oder Kassascheine des Bundes werden zu einem zu bestimmenden Kurse an Zahlungsstatt genommen.
    Durch Bundesgesetz wird bestimmt, ob und unter welchen Bedingungen Obligationen von Kantonen und Gemeinden und andere Vermögenswerte an Zahlungsstatt genommen werden.
    Ebenso kann der Abgabepflichtige verpflichtet werden, Wertpapiere und andere Vermögenswerte an Zahlungsstatt abzuliefern.
    Die Fälle dieser Naturalabgabe wie die Bewertungsgrundsätze werden durch Bundesgesetz festgelegt.
  14. Veranlagung und Bezug der Vermögensabgabe erfolgt nach Weisung und unter Aufsicht des Bundes durch die Kantone. Die Kosten werden von Bund, Kantonen und Gemeinden entsprechend ihrem Anteil am Ertrag der Vermögensabgabe getragen.
  15. Die Bundesversammlung stellt nach Annahme des Verfassungsartikels durch dringlichen Bundesbeschluss diejenigen Vorschriften auf, welche eine volle steuerliche Erfassung des in Wertpapieren liegenden Vermögens sichern und die Kapitalflucht ins Ausland verhindern.
    Auf einen bestimmten Termin ist namentlich die Abstempelung der Wertpapiere durch den Staat zu ordnen. Bei Wertpapieren, die der Abstempelung entzogen werden, erlischt die Zahlungspflicht des betreffenden Schuldners.
  16. Die Selbsttaxation ist obligatorisch.
    Alle natürlichen und juristischen Personen sind der Steuerbehörde gegenüber zur Auskunft verpflichtet. Insbesondere sind die Geldinstitute verpflichtet, sich allen Kontrollmassnahmen der Einschätzungsorgane zu unterziehen.
  17. Unter welchen Voraussetzungen eine Revision der Einschätzung erfolgen kann, bestimmt das Gesetz.
  18. Die Kantone und die Gemeinden erhalten je 20 vom Hundert der in ihrem Gebiet eingehenden Abgabebeträge, Nachsteuern, Zinsen und Bussen. Die übrigen 60 vom Hundert fallen dem Bund zu.
  19. Nach Erhebung der einmaligen Vermögensabgabe tritt dieser Verfassungsartikel wieder ausser Kraft.
  Für juristische Personen beträgt die Vermögensabgabe 10 vom Hundert des abgabepflichtigen Vermögens.

## Footnotes

1 Im Wahlprogramm der Grünen in Deutschland fand sich die Forderung nach einer Vermögensabgabe, um die Folgekosten der Finanzkrise zu tragen (vgl. Bach et al. 2010). Institutionen wie der Internationale Währungsfonds (International Monetary Fund 2013, S. 49) und die Deutsche Bundesbank (2014, S. 52–54) reagierten auf derartige Forderungen.

2 Siehe Bach (2020) für eine ausführliche Beschreibung der Forderung.

3 Als Teilevidenz für diese Erwartungshaltung mag dienen, dass Forderungen nach „einmaligen“ Vermögensabgaben nach vielen größeren und kleineren Krisen in kurzen Zeitintervallen erhoben werden.

4 Eine verfassungsrechtliche Abhandlung, unter welchen Umständen eine einmalige Vermögensabgabe in welcher Form überhaupt eingehoben werden kann, liegt außerhalb der Fragestellung dieses Beitrages.

5 Reckendrees (2004) untersucht die ökonomische Tragfähigkeit der deutschen Lastenausgleichsabgabe und findet, dass die damaligen makroökonomischen Rahmenbedingungen die Vermögensabgabe in Summe tragfähig machten.

6 Der Aktienwert von Tui AG lag trotz leichter Erholung Anfang November noch knapp 2/3 unter dem Wert zu Beginn des Jahres.

7 Der aktuelle Vorschlag der Partei DIE LINKE, wie in der Auftragsstudie von Bach (2020) dargelegt, bezieht sich auf die Vermögensabgabe im deutschen Lastenausgleich von 1952 und sähe eine Bezahlung über einen Zeitraum von 20 Jahren vor. Eine einmalige Vermögensabgabe in Höhe von 30 % welche im Laufe von 20 Jahren zu bezahlen ist, würde bei einem nominellen Nachsteuerertrag von 7 % p. a. und einer Inflationsrate von 3,5 % einen realen Vermögenszuwachs von ca. 60 % in 20 Jahren erlauben. Bei einem nominellen Nachsteuerertrag von 2,5 % p. a. und einer Inflationsrate von 1,5 % würde sich das reale Vermögen um ca. 7 % in 20 Jahren reduzieren.

8 Die Umrechnung erfolgte auf Basis des Wachstums des nominalen Bruttoinlandsprodukts pro Kopf nach Daten von Boris Zürcher (2010) von 1920 bis 2008.

9 Originalwortlaut: „Nach Erhebung der einmaligen Vermögensabgabe tritt dieser Verfassungsartikel wieder ausser Kraft“ (vgl. Anhang mit Text der Initiative).

10 Zum damaligen Zeitpunkt waren in der Schweiz nur Männer stimmberechtigt.

11 Die graphische Darstellung der Ergebnisse auf Bezirksebene beinhaltet minimale Unschärfen, da die Ergebnisse von 1922 den geographischen Bezirken auf aktuellem Stand zugeordnet werden.

12 Die Daten stammen aus dem Statistischen Jahrbuch für die Schweiz, herausgegeben vom Bundesamt für Statistik (1922).

13 Die geringe Zahl an Beobachtungen und die historische Natur der Daten erlauben keine Analyse, die potenziell vorhandene Endogenität berücksichtigen könnten. Daher interpretieren wir die Resultate als nicht kausal.

14 Der Kanton Tessin hat mit ca. 65 % als einziger Kanton eine Wahlbeteiligung unter 80 %.

15 Mehrere erklärende Variablen sind durch Ausreißer gekennzeichnet. So weist zum Beispiel der Kanton Basel-Stadt eine um das Vielfache höhere Bevölkerungsdichte als alle anderen Kantone aus und der Kanton Neuenburg weist für das Jahr 1922 eine massive hohe Zahl an Arbeitssuchenden aus.

16 So waren beispielsweise 1922 keine Frauen bei der Abstimmung beteiligt. Stutzer und Kienast (2005) zeigen, dass die Einführung des Frauenstimmrechts in der Schweiz einen konservativen Einfluss auf das Ausgabenwachstum in den Kantonen hatte. Eine Senkung des Ausgabenwachstums aufgrund des Frauenstimmrechts würde gehen eine größere Zustimmung zu neuen Abgaben – und damit einer Ausweitung des Staatsbudgets – sprechen.
